# Medical College Notes

**Published:** 1894-09

**Authors:** 


### Medical College Notes.

The College of Physicians and Surgeons, St. Louis, announces a series of lectures by eminent American physicians and teachers. Among the non-resident lecturers announced are the following: Nicholas Senn, of Chicago, will give a series of lectures and clinics on surgery; Joseph Price, of Philadelphia, will lecture upon the technique of abdominal section; Joseph M. Mathews, of Louisville, will deliver lectures upon diseases of the rectum; and Joseph Eastman, of Indianapolis, will speak on the subject of hysterectomy. Besides these, William Porter, of St. Louis, will give a course of lectures on physical diagnosis, and James Moores

Ball, of St. Louis, will give five or six lectures on the history of medicine. It is expected that other lectures by well-known physicians will be announced later. This special didactic series will be given on Thursday evenings, and there is no extra fee charged to regular matriculates of the college.

This is an excellent plan, and distinguished physicians from various portions of the country should be oftener invited by the faculties of medical schools to address their students. Such a fashion enlarges the scope of training and enriches the curriculum.

---

THE forty-ninth regular session of the Medical Department of the University of Buffalo will begin on Monday, September 24, 1894. The following changes in the faculty and teaching corps have occurred : Dr. Arthur W. Hurd has been promoted from lecturer to professor on insanity ; Dr. Francis T. Metcalfe has been appointed lecturer on comparative pathology ; Dr. Ferdinand G. Moehlau has been appointed instructor in physiology, and Dr. J. F. Whitwell instructor in general pathology. The following clinical instructors have been added to the staff : On surgery, Dr. J. F. Whitwell ; diseases of the nervous system, Dr. James A. Gibson ; diseases of children, Dr. Maud J. Frye ; diseases of the skin, Dr. Grover Wende ; diseases of the nose and throat, Dr. George F. Cott.

---

THE twelfth annual college year of the Medical Department of Niagara University will open on Tuesday, September 25, 1894. The changes of teachers during the year have been as follows : Dr. Earl P. Lothrop has been appointed clinical assistant in obstetrics ; Dr. Alfred E. Diehl, lecturer on histology ; Dr. George Roberts, lecturer on chemistry, and Dr. Robert A. Poynton, lecturer on anatomy. Dr. L. Bradley Dorr has been promoted from lecturer on, to professor of, bacteriology.

